# Dyspnea as a marker of prognosis in immunocompromised patients with acute respiratory failure

**DOI:** 10.1016/j.aicoj.2026.100091

**Published:** 2026-05-19

**Authors:** Alexandre Demoule, Maxens Decavèle, Sangeeta Mehta, Philippe R. Bauer, Victoria Metaxa, Frédéric Pène, Christophe Girault, Laveena Munshi, Fabio Silvio Taccone, Massimo Antonelli, Francois Barbier, Andreas Barratt-Due, Gaston Burghi, Emmanuel Canet, Achille Kouatchet, Virginie Lemiale, Ignacio Martin-Loeches, Djamel Mokart, Anne-Sophie Moreau, Luca Montini, Peter Pickkers, Jordi Rello, Peter Schellongowski, Nicolas Terzi, Miia Valkonen, Andry van de Louw, Elie Azoulay, Michael Darmon, for the Efraim investigators and the Nine-I study group

**Affiliations:** a AP-HP. Sorbonne Université, Hôpital Pitié-Salpêtrière, Service de Médecine Intensive – Réanimation (Département "R3S"), Paris, France; b Sorbonne Université, INSERM, UMRS1158 Neurophysiologie Respiratoire Expérimentale et Clinique, Paris, France; c Department of Medicine, Sinai Health System, Interdepartmental Division of Critical Care Medicine, University of Toronto, Toronto, Ontario, Canada; d Division of Pulmonary and Critical Care Medicine, Mayo Clinic, Rochester, MN 55905, United States of America; e Department of Critical Care, King's College Hospital NHS Foundation Trust, London SE5 9RS, United Kingdom; f Université Paris Cité, Assistance Publique – Hôpitaux de Paris, Hôpital Cochin, DMU Réanimation-Urgences, Service de Médecine Intensive Réanimation, Institut Cochin, INSERM U1016, CNRS UMR8104, Paris, France; g Medical Intensive Care Unit, CHU Rouen, and Univ Rouen Normandie, Normandie Univ, GRHVN-UR 3830, F-76000 Rouen, France; h Department of Intensive Care, Hôpital Universitaire de Bruxelles (HUB), Université Libre de Bruxelles (ULB), Brussels, Belgium; i Department of Anesthesiology, Intensive Care and Emergency Medicine, Fondazione Policlinico Universitario A.Gemelli IRCCS, Rome, Italy; j Medical Intensive Care Unit, La Source Hospital, CHR Orléans, Orléans, France; k Department of Anesthesia and Intensive Care, Division of Emergencies and Critical Care, Oslo University Hospital, Oslo, Norway; l Intensive Care Unit, Hospital Maciel, Montevideo, Uruguay; m Medecine Intensive Reanimation, Nantes University Hospital and Nantes Université, Nantes, France; n Department of Medical Intensive Care Medicine, University Hospital of Angers, Angers, France; o Medecine Intensive et Réanimation, AP-HP, Hôpital Saint-Louis, Paris, France; p Trinity College Dublin, School of Medicine, Dublin, Ireland; q Département d'Anesthésie-Réanimation, Institut Paoli-Calmettes, Marseilles, France; r Médecine Intensive et Réanimation, Centre Hospitalier Universitaire de Lille, Lille, France; s Catholic University of Sacred Heart, Rome, Italy; t The Department of Intensive Care Medicine (710), Radboud University Medical Center, Nijmegen, the Netherlands; u Medicine Department, Universitat Internacional de Catalunya, Spain; v Clinical Research Pneumonia and Sepsis (CRIPS) Research Group-Vall d'Hebron Institute Research (VHIR), Barcelona, Spain; w Centro de Investigación Biomédica en Red de Enfermedades Respiratorias (CIBERES), Instituto Salud Carlos III, Madrid, Spain; x Department of Medicine I, Medical University of Vienna, Vienna, Austria; y Medical Intensive Care Unit, University Hospital of Rennes, Rennes, France; z Division of Intensive Care Medicine, Department of Anesthesiology, Intensive Care and Pain Medicine, University of Helsinki and Helsinki University Hospital, Helsinki 00014, Finland; aa Division of Pulmonary and Critical Care Medicine, Penn State Health Milton Hershey Medical Center, Hershey, PA 17036, United States of America; ab ICU-People Research Team, INSERM 1342, Institut de Recherche Saint-Louis, Paris, France; ac INSERM 1342, Institut de Recherche Saint-Louis, Paris, France

**Keywords:** Immunocompromised, High flow oxygen, Non-invasive ventilation, Mechanical ventilation, Acute respiratory failure, Dyspnea, Comfort, Intubation, Diagnosis, Outcome.

## Abstract

**Background:**

Acute hypoxemic respiratory failure (AHRF) is the leading cause of intensive care unit (ICU) admission in immunocompromised patients, in whom both intubation and mortality rates are higher than in the general ICU population. This study explores dyspnea on admission as it relates to clinical outcomes.

**Methods:**

Secondary analysis of the Efraim study, a prospective multinational cohort study of immunocompromised patients with AHRF admitted to the ICU. Dyspnea was quantified by a numeric rating scale (dyspnea-NRS) from zero to 10. Factors associated with dyspnea-NRS were assessed with linear regression. Hierarchical model was used to assess factors independently associated with invasive mechanical ventilation (intubation) and hospital mortality.

**Results:**

547 patients were included. On ICU admission, median dyspnea-NRS was 5 (interquartile range 4–7). Variables independently associated with dyspnea-NRS were underlying immune defect unrelated to hematological malignancy, chronic heart failure, high SOFA score and respiratory rate. Intubation rate was 41 %. Variables independently associated with intubation were dyspnea-NRS ≥5 (odds ratio [OR] 2.61, p < 0.001), high SOFA (OR per point 1.10, p = 0.006) and fungal infection (OR 2.02, p = 0.020)., while respiratory rate and PaO_2_/FiO_2_ were not. Hospital mortality was 37 %. Variables independently associated with hospital mortality were age (OR per year 1.02, P = 0.009), SOFA score (OR per point, 1.13, P < 0.001) and dyspnea-NRS (OR per point 1.19, P < 0.001).

**Conclusions:**

In immunocompromised patients admitted to the ICU for AHRF, dyspnea at admission is moderate to severe and is associated with clinical outcomes. Dyspnea-NRS ≥5 is associated with an increase in intubation rate and hospital mortality.

## Introduction

Acute hypoxemic respiratory failure (AHRF) is the leading cause of intensive care unit (ICU) admission in immunocompromised patients and a severe complication with a mortality that can reach 60 % [1,2]. With the growing number of immunocompromised adults, encounters with patients presenting with AHRF are expected to become increasingly frequent [3].

Dyspnea, defined as “the symptom that conveys an upsetting or distressing awareness of breathing”, is a key clinical feature of AHRF [4]. Unlike physical signs of AHRF like tachypnea, labored breathing or cyanosis, which clinicians may observe and quantify, dyspnea is a symptom, which places a very strong emphasis on self-reporting, much like pain [4]. However, as opposed to pain, dyspnea has not received major attention. First, because it results from the respiratory system load capacity imbalance, dyspnea is a warning sign. In unselected populations of patients admitted to the ICU for AHRF and receiving either standard oxygen, non-invasive ventilation or high flow nasal cannula, severe dyspnea is associated with increased intubation rate and mortality [[5], [6], [7]]. Second, being a noxious sensation, dyspnea is described by patients as one of the most distressing experience of their ICU stay [8,9]. As a consequence, dyspnea contributes to the pathogenesis of post-traumatic stress disorder in ICU survivors [4]. Given the high prevalence of AHRF and its severity in immunocompromised patients, data on dyspnea in this specific population would be of the upmost interest. Dyspnea may help improving the management of immunocompromised patients with AHRF, for instance the intubation decision making process.

Here, we hypothesized that, in non-intubated immunocompromised patients managed for AHRF, dyspnea severity on ICU admission is associated with higher risk of intubation and mortality. The primary aim was to assess whether dyspnea severity on admission is associated with the need for intubation. Secondary aims were to identify factors associated with higher dyspnea severity and to evaluate the association between dyspnea severity and hospital mortality.

## Methods

This is a preplanned secondary analysis of the Efraim multinational, observational prospective cohort study on immunocompromised patients admitted in the ICU for AHRF. This initiative from the Nine-I (Caring for critically ill immunocompromised patients) study group has included patients from 68 ICUs in 16 countries. Participating physicians and teams have extensive experience in the management of various groups of critically ill immunocompromised patients. The full Efraim protocol and results have been published elsewhere [2]. Participating centers and collaborators are listed in the acknowledgements. The study was approved by the institutional review board of each institution in accordance with local ethics regulation (Table E1 in the Online Supplement). We followed the STROBE guidelines for the reporting of observational studies [10] (Table E2 in the Online Supplement).

### Study population

After IRB approval, each participating ICUs included patients between November 2015 and July 2016. Inclusion criteria were age ≥18 years, acute hypoxemic respiratory failure (PaO_2_ < 60 mmHg or SpO_2_ <90% on room air, or tachypnea >30/min, or labored breathing or respiratory distress or dyspnea at rest or cyanosis), need for more than 6 L/min oxygen, respiratory symptom duration less than 72 h and non-AIDS-related immune deficiency defined as hematologic malignancy or solid tumor (active or in remission for less than 5 years, including recipients of hematopoietic cell transplantation), solid organ transplant, long-term (>30 days) or high-dose (>1 mg/kg/day) steroids, or any immunosuppressive drug for more than 30 days. Patients with postoperative acute respiratory failure (within 6 days of surgery), those admitted after a cardiac arrest, patients admitted only to secure bronchoscopy, and patients/surrogates who declined study participation were not included.

For the purposes of the present secondary analysis, only patients who were not intubated at the time of ICU admission were included. Patients with missing data regarding dyspnea severity were excluded.

### Data collection

Study investigators completed a standardized paper case report form that was eventually sent to the coordinating center in Paris.

Demographic data and medical history collected consisted of: age, gender, body mass index, performance status, respiratory or cardiac comorbidity, cause of immunosuppression and neutropenia. The precipitating factor of AHRF was recorded. Data on the current AHRF episode included Sequential Organ Failure Assessment score (SOFA) [11], respiratory rate, respiratory comfort, chest radiography, arterial blood gas and initial oxygenation strategy. Dyspnea, termed “comfort with breathing” on the case report form, was assessed by investigators with a patient self-reported numerical rating scale (dyspnea-NRS) from zero (no respiratory discomfort) to 10 (worst possible respiratory comfort). Of note, this question did not target discomfort associated with the respiratory interface (mask, nasal canulae, etc.) or the discomfort associated with any non-invasive respiratory support delivered to the patient (non-invasive ventilation, high flow nasal canulae, etc.). No trigger question regarding dyspnea absence or presence (yes-no question) was asked to the patients. Dyspnea was assessed once, on the day of admission. Need for intubation (intubation criteria were not predefined), catecholamine and renal replacement therapy was recorded, as well as occurrence of Acute Respiratory Distress Syndrome according the Berlin definition [12]. ICU, in-hospital and 90-day mortality were recorded, as well as ICU and hospital length of stay. Patient code status on ICU admission (full code or treatment limitation decision) was recorded.

### Outcomes

The primary outcome was intubation during ICU stay. Secondary outcomes were ICU, hospital and 90-day mortality, as well as ICU and hospital length of stay.

### Statistical analysis

Quantitative variables were described as median (interquartile range [IQR]) and were compared between groups using the non-parametric Wilcoxon rank-sum (two groups) test or Kruskal-Wallis test (more than two groups). Qualitative variables were described as frequency (percentages) and were compared between groups using Fisher’s exact test.

Dyspnea-NRS on admission was used to identify four groups of patients according to quartile: zero to 3, 4 to 5, 6 to 8 and 9 to 10. We chose these cut-offs because the present study is exploratory and because there are very few data on dyspnea in non-intubated patients [[5], [6], [7]]. The commonly accepted cut-off of 3 or 4 is used to define moderate to severe dyspnea intubated patients [4]. Hierarchical models were used to assess factors independently associated with dyspnea, intubation and mortality. To assess variables associated with dyspnea severity on ICU admission, a correlation plot was performed between dyspnea and continuous variables. Then a linear regression was performed, dyspnea being the variable of interest. Conditional stepwise regression with 0.2 as the critical P-value for entry into the model, and 0.1 as the P-value for removal. Dyspnea reporting being liable to vary from center to center, the final model was a mixed model with center as a random effect on the intercept.

Logistic regression was performed to assess variables associated with need for intubation over ICU stay and with hospital mortality. To assess linear relationship of dyspnea with invasive ventilation and mortality, a gam model was performed and resulting spline is reported. Based on the spline, we decided to analyze association between dyspnea and mechanical ventilation as a binary variable (dyspnea-NRS <5 vs. ≥5) and association of dyspnea with mortality as a continuous variable.

We used conditional stepwise regression with 0.2 as the critical P-value for entry into the model, and 0.1 as the P-value for removal. It was planned a priori to force dyspnea in the model should this variable not be selected. Interactions and correlations between the explanatory variables were carefully checked. Continuous variables for which log-linearity was not confirmed were transformed into categorical variables according to median or IQR. The final models were assessed by calibration, discrimination and relevancy. Residuals were plotted, and the distributions inspected. A hierarchical model was then performed using variables previously selected along with center as random effect on the intercept. This model adjusting for clustering effect was planned a priori to be main result of the analysis. Same validation methods were used as previously. Adjusted odds ratios (OR) of variables present in the final model are presented with their 95% confidence intervals (CI).

A p value <0.05 was considered significant. Statistical analyses were performed with IBM SPSS Statistics, version 20.0 (IBM SPSS Inc., Chicago, IL, USA) and with R statistical software, version 3.4.4 (available online at http://www.r-project.org/) and packages ‘mgcv’, ‘survival’, ‘lme4’, and ‘lmerTest’.

## Results

### Study population

**Fig. 1:**
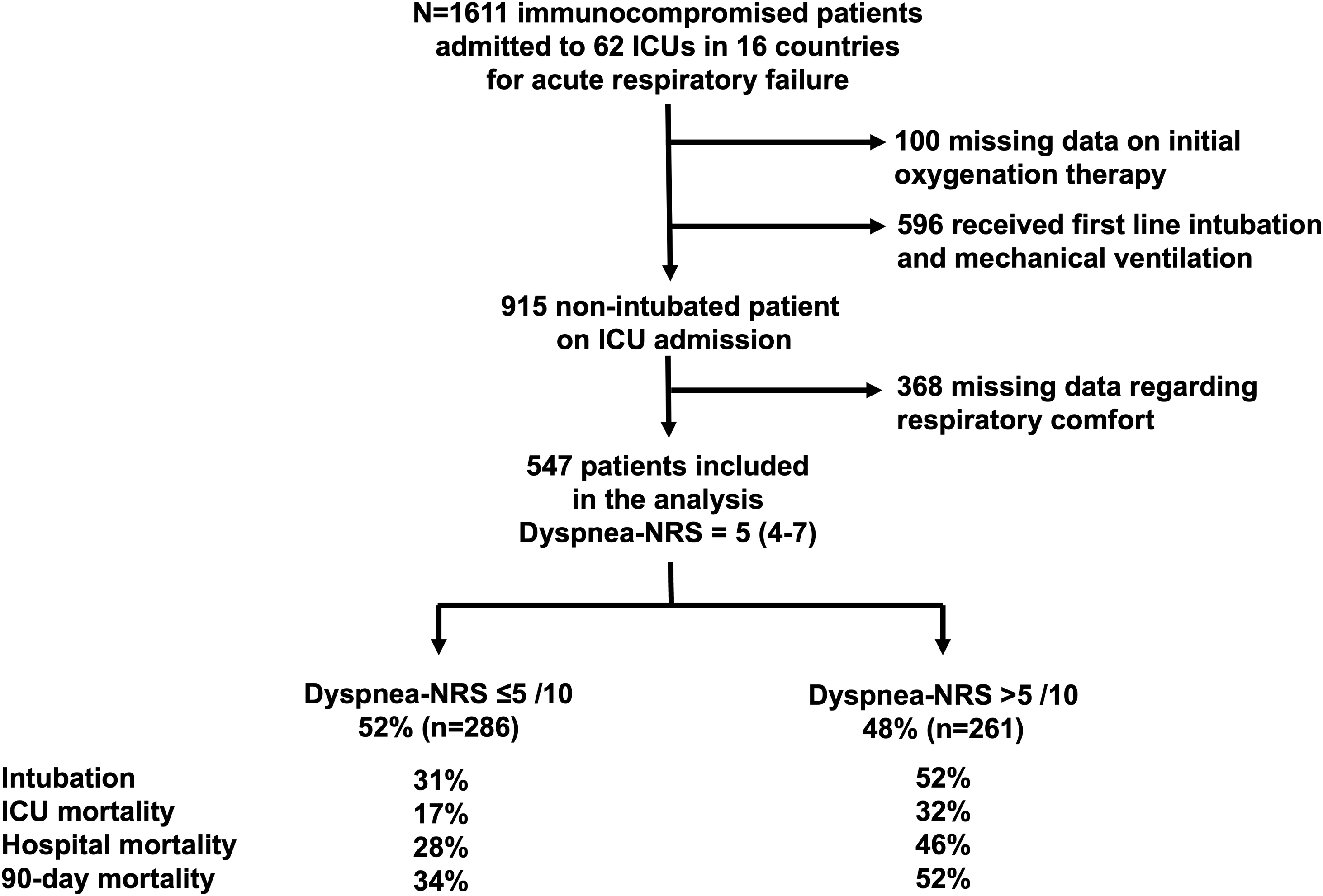
Flow chart of the study. ICU, intensive care unit; dyspnea-NRS, numeric rating scale for dyspnea.

During the study period, 1611 immunocompromised patients were admitted for AHRF, among whom 596 were intubated on ICU admission. Data on initial respiratory support were missing in 100. Among the 915 patients who were not intubated on ICU admission, dyspnea was not recorded in 368 patients. A total of 547 patients (60% of non-intubated patients) were included in this analysis (Fig. 1). Table 1 displays the main characteristics of these patients.

**Table 1:** Characteristics at the time of inclusion according to dyspnea intensity on the day of admission.

	All n = 547		Dyspnea-NRS 0−3 n = 130	Dyspnea-NRS 4−5 n = 156	Dyspnea-NRS 6−8 n = 185	Dyspnea-NRS 9−10 n = 76	p
Patient characteristics							
Female sex*, n (%)*	202 (37)		47 (36)	52 (33)	70 (38)	33 (44)	0.462
Age, *years*	64 (54–71)		64 (55–71)	64 (52–71)	64 (55–70)	65 (55–72)	0.809
BMI *kg.m^−2^*	25 (22–29)		25 (20–28)	25 (21–29)	24 (21–29)	24 (22–28)	0.918
COPD*, n (%)*	86 (16)		20 (16)	8 (15)	31 (19)	27 (14)	0.727
Chronic heart failure*, n (%)*	130 (25)		21 (16)	35 (22)	43 (25)	31 (41)	0.001
Poor performance status^a^ (ECOG ≥ 2)*, n (%)*	224 (46)		45 (41)	65 (46)	72/161 (45)	42/70 (60)	0.106
Underlying condition, n (%)							
Hematological malignancy	334 (61)		77 (59)	98 (63)	116 (63)	43 (57)	0.925
Solid tumor	167 (31)		47 (36)	46 (30)	46 (25)	28 (37)	0.100
Systemic diseases	85 (16)		12 (9)	24 (15)	32 (17)	17 (23)	0.039
Solid organ transplant	35 (6)		8 (7)	11 (7)	11 (7)	5 (7)	0.987
Allogenic HCT	101 (18)		21 (16)	32 (21)	34 (18)	13 (17)	0.329
AHRF episode							
Duration of symptoms prior to admission, *days*	2 (1–7)		2 (1–7)	2 (1–6)	3 (1–8)	3 (1–7)	0.366
SOFA	6 (3–8)		5 (3–7)	6 (3–9)	6 (4–9)	7 (4–9)	0.001
*Cause of AHRF, n (%)*							0.174
Bacterial pneumonia	118 (22)		30 (23)	37 (24)	36 (20)	15 (20)	
Viral pneumonia	77 (14)		10 (8)	29 (19)	32 (17)	6 (8)	
Invasive fungal disease	66 (12)		18 (14)	15 (10)	28 (15)	5 (7)	
Disease related infiltrates	79 (14)		20 (15)	18 (12)	24 (13)	17 (22)	
Cardiogenic pulmonary edema	45 (8)		10 (8)	13 (8)	13 (7)	9 (12)	
Other	93 (17)		26 (20)	27 (17)	26 (14)	14 (18)	
Undetermined	69 (13)		16 (13)	17 (11)	26 (14)	10 (13)	
At ICU admission							
Respiratory rate, *min^−1^*		32 (27–37)	28 (23–35)	30 (26–35)	34 (28–38)	35 (30–40)	<0.001
Bilateral alveolar infiltrate on chest x-ray*, n (%)*		202 (37)	35 (27)	52 (33)	84 (45)	31 (41)	0.007
PaO_2_/FiO_2_, *mmHg*		169 (118–265)	186 (121–274)	168 (118–241)	169 (124–273)	155 (103–290)	0.359
PaCO_2_, *mmHg*		35 (29–43)	33 (29–39)	35 (28–42)	36 (30–43)	38 (31–55)	0.008
Neutropenia*, n (%)*		113 (21)	21 (16)	35 (22)	42 (23)	15 (20)	0.490

Quantitative variables are reported as median (interquartile range) and qualitative variables are reported as frequency (percentages).

NRS, dyspnea numerical rating scale; BMI, body mass index; COPD, chronic obstructive pulmonary disease; ECOG, eastern oncology study group; HCT, hematopoietic cells transplantation; AHRF, acute hypoxemic respiratory failure; SOFA, Sequential Organ Failure Assessment score.

a ECOG was available in 484 patients.

Age was 64 (54–71) years, with 37% women. Median SOFA score was 6 (3–8). The underlying condition was a hematological malignancy in 334 patients (61%), a solid tumor in 167 (31%), a systemic disease in 85 (16%, among whom 65% were receiving high or low-dose corticosteroids) and a solid organ transplant in 35 (6%). One hundred and one (18 %) patients received hematopoietic cells transplantation.

Cause of AHRF was bacterial pneumonia, in 118 patients (22%), viral pneumonia in 77 (14%), invasive fungal infection in 66 (12%), disease related infiltrates in 79 (14%), acute cardiogenic pulmonary edema in 45 (8%), other in 93 (17%, including toxic pneumonia, lower respiratory tract airway disease and extrapulmonary sepsis) and undetermined in 69 patients (13%).

On ICU admission, median dyspnea-NRS was 5 (4–7). Dyspnea-NRS was ≥3 in 457 patients (84%) and was ≥4 in 417 patients (76%). Table 1 shows main patients characteristics according to quartile of dyspnea-NRS.

### Association between dyspnea severity and intubation

**Fig. 2:**
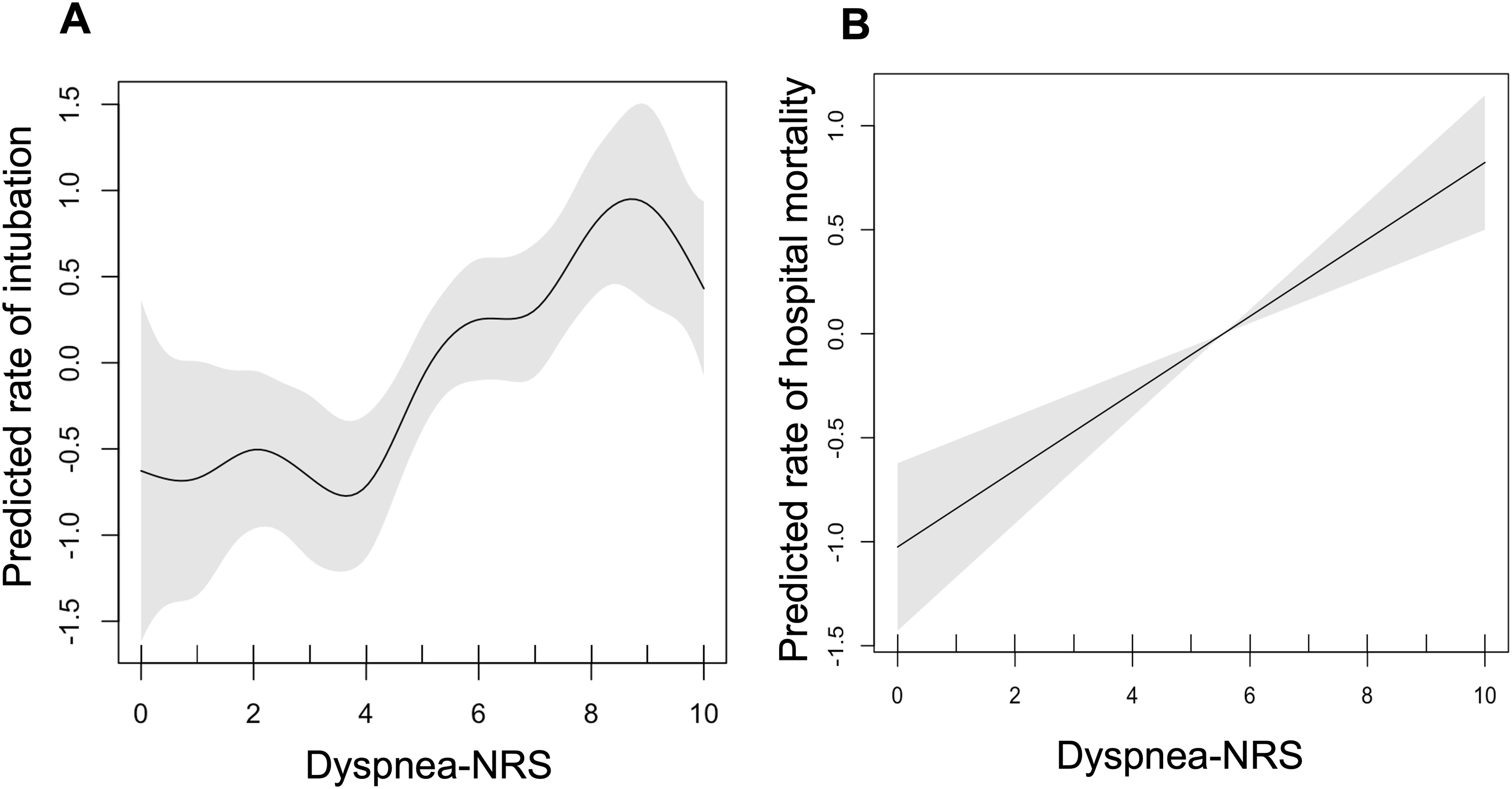
Restricted cubic splines showing predicted rate of intubation (A) and mortality (B) across dyspnea-numerical rating scale (dyspnea-NRS) at admission.

The intubation rate was 41 % (n = 226). There was a biphasic association between dyspnea and the risk of intubation, (Fig. 2, Panel A), the risk of intubation being constant and low in patients with dyspnea-NRS <5 and then increasing steadily when dyspnea-NRS is ≥5.

The comparison between patients not intubated and intubated is shown in Table 3. After adjustment for confounders and clustering effect, three variables were independently associated with intubation: dyspnea-NRS ≥5 (OR 2.61, 95% CI 1.74–3.91, P < 0.001), SOFA (OR per point 1.10, 95% CI 1.04–1.17, P = 0.006), and AHRF etiology, with fungal infection being associated with a higher risk of intubation (OR 2.02, 95% CI 1.03–3.96, P = 0.02, bacterial infection as reference etiology). When forced into the final model, respiratory rate, PaO_2_/FiO_2_ and the type of respiratory support were neither selected nor changed the final model.

After intubation, PaO_2_/FiO_2_ was lower in patients with dyspnea-NRS ≥5 on ICU admission (130 [100–195] mmHg vs. 190 [105–255] mmHg, P = 0.001). Patients with dyspnea-NRS ≥5 on ICU admission were more likely to develop moderate or severe acute respiratory distress syndrome after intubation (73 % vs. 50 %, P = 0.02).

### Association between dyspnea severity and mortality

ICU mortality was available in all patients. Hospital mortality and 90-day mortality were available in 522 and 452 patients, respectively. ICU, hospital and 90-day mortality were 24 %, 37 % and 42 %, respectively. On univariate analysis, higher dyspnea-NRS was associated with higher ICU, hospital and 90-day mortality (Table 2). Dyspnea was not associated with ICU or hospital length of stay and was not associated with the use of vasopressors or renal replacement therapy after intubation (Table 2).

**Table 2:** Management and outcome according to dyspnea intensity on the day of admission.

	All n = 547	Dyspnea-NRS 0−3 n = 130	Dyspnea-NRS = 4−5 n = 156	Dyspnea-NRS = 6−8 n = 185	Dyspnea-NRS = 9−10 n = 76	p
Non-invasive respiratory support, n (%)						<0.001
Conventional oxygen therapy	288 (53)	84 (65)	74 (47)	92 (50)	38 (50)	
Non-invasive ventilation (NIV)	85 (16)	11 (8)	18 (12)	33 (18)	23 (30)	
High flow oxygen (HFO)	109 (20)	22 (17)	41 (26)	36 (20)	10 (13)	
NIV + HFO	65 (12)	13 (12)	23 (15)	24 (13)	5 (7)	
Advanced life support, n (%)						
Intubation	226 (41)	37 (28)	52 (33)	95 (51)	42 (55)	<0.001
Vasopressors	240 (44)	57 (44)	63 (42)	83 (45)	37 (49)	0.669
Renal replacement therapy	70 (13)	14 (11)	13 (8)	32 (17)	11 (15)	0.078
Outcome, n (%)						
ICU mortality	134 (24)	18 (14)	32 (21)	57 (31)	27 (36)	<0.001
Hospital mortality^a^	201 (39)	30 (25)	50 (34)	81 (46)	40(53)	<0.001
90-day mortality^b^	232 (51)	38 (41)	59 (47)	91 (57)	44 (61)	0.022
ICU length of stay, *days*	8 (4–15)	7 (4–13)	7 (4–16)	8 (5–14)	8 (4–17)	0.624
Hospital length of stay, *days*	25 (14–41)	23 (15–42)	27 (15–46)	25 (113‒39)	26 (12–36)	0.635

Quantitative variables are reported as median (interquartile range [IQR]) qualitative variables are reported as frequency (percentages).

NRS, dyspnea numerical rating scale; ICU, intensive care unit;

a Hospital mortality was available in 522 patients and ^b^90-day mortality was available 452 patients.

**Table 3:** Factors associated with intubation by univariate analysis.

	Univariate analysis			Multivariate analysis	
	Patients not intubated n = 321	Patients intubated n = 226	p	Odds ratio (95% confidence interval)	p
Patients characteristics					
Female sex*, n (%)*	116 (36)	86 (38)	0.667		
Age, *years*	65 (55–72)	63 (51–70)	0.037		
BMI, *kg. m^−2^*	26 (22–29)	25 (23–29)	0.423		
COPD*, n (%)*	60 (20)	26 (12)	0.028		
Chronic heart failure*, n (%)*	76 (24)	54 (24)	0.733		
Poor performance status^a^ (ECOG ≥ 2)*, n (%)*	128 (40)	96 (42)	0.470		
*Underlying condition, n (%)*					
Hematological malignancy	198 (62)	136 (60)	0.722		
Solid tumor	103 (32)	64 (28)	0.346		
Systemic diseases	42 (13)	43 (19)	0.059		
Solid organ transplant	18 (6)	17 (8)	0.300		
Allogenic HCT	51 (16)	50 (22)	0.064		
AHRF episode					
Duration of symptoms prior to admission, *days*	2 (1–7)	3 (1–8)	0.103		
SOFA	5 (3–8)	6 (4–9)	0.002	1.10 (1.04–1.17)	0.006
*Cause of AHRF, n (%)*			0.005		
Bacterial pneumonia	74 (23)	44 (20)		Ref	
Viral pneumonia	42 (13)	35 (16)		1.16 (0.59−2.23)	0.666
Invasive fungal disease	30 (9)	36 (16)		2.02 (1.03–3.96)	0.02
Disease related infiltrates	48 (15)	31 (14)		0.88 (0.45−1.69)	0.694
Acute cardiogenic pulmonary edema	31 (10)	14 (6)		0.56 (0.25−1.24)	0.151
Other	65 (20)	28 (12)		0.61 (0.32−1.6)	0.131
Undetermined	31 (10)	38 (17)		1.45 (0.74−2.84)	0.280
At ICU admission					
Respiratory rate, *min^−1^*	31 (26–36)	33 (28–38)	0.003		
Dyspnea-NRS	5 (3–7)	6 (5–8)	<0.001		
Dyspnea ≥5*, n (%)*	184 (57)	178 (79)	<0.001	2.61 (1.74–3.91)	<0.001
Bilateral alveolar infiltrate on chest x-ray*, n (%)*	89 (28)	113 (50)	<0.001		
PaO_2_/FiO_2_, *mmHg*	178 (124–271)	151 (111–240)	0.013		
PaCO_2_, *mmHg*	35 (29–43)	36 (29–44)	0.204		
Neutropenia*, n (%)*	57 (18)	56 (25)	0.046		
Non-invasive respiratory support			0.384		
Conventional oxygen therapy*, n (%)*	170 (53)	118 (52)			
Non-invasive ventilation (NIV)*, n (%)*	56 (17)	29 (13)			
High flow oxygen (HFO)*, n (%)*	60 (19)	49 (22)			
NIV and HFO*, n (%)*	30 (9)	65 (29)			

Quantitative variables are reported as median (interquartile range [IQR]) qualitative variables are reported as frequency (percentages).

BMI, body mass index; COPD, chronic obstructive pulmonary disease; ECOG, eastern oncology study group; HCT, hematopoietic cells transplantation; AHRF, acute hypoxemic respiratory failure; SOFA, Sequential Organ Failure Assessment score; ICU, intensive care unit; dyspnea-NRS, numeric rating scale for dyspnea.

a ECOG was available in 484 patients.

The association between dyspnea and hospital mortality was linear (Fig. 2B). The comparison between survivors and non survivors at hospital discharge is shown in Table 4. After adjustment for confounders and clustering effect, three variables were independently associated with higher hospital mortality: age (OR per year 1.02, 95%CI 1.00–1.03; P = 0.009), severity as assessed by the SOFA score (OR per point, 1.13, 95%CI 1.07–1.20; P < 0.0001) and dyspnea-NRS (OR per point 1.19, 95%CI 1.10–1.29; P < 0.0001). When forced in the final model, performance status, the cause of AHRF and the type of respiratory support were neither selected nor changed the final model. In a sensitivity analysis, dyspnea-NRS remained independently associated with ICU mortality ((OR per point 1.22, 95%CI 1.11–1.34; P < 0.0001).

**Table 4:** Univariate analysis: Factors associated with in-hospital mortality (n = 522).

	Univariate analysis			Multivariate analysis	
	Survivors n = 321	Non-survivors n = 201	p	Odds ratio (95% confidence interval)	p
Patient characteristics					
Female sex*, n (%)*	122 (38)	72 (36)	0.596		
Age, *years*	63 (53–71)	66 (56–74)	0.026	1.02 (1.00–1.03)	0.009
Poor performance status^a^ (ECOG ≥ 2)*, n (%)*	112 (39)	118 (59)	<0.001		
BMI, *kg.m^−2^*	25 (22–29)	25 (23–29)	0.465		
COPD*, n (%)*	53 (17)	26 (13)	0.348		
Chronic heart failure*, n (%)*	75 (23)	51 (25)	0.466		
*Underlying condition, n (%)*					
Hematological malignancy	190 (59)	127 (63)	0.363		
Solid tumor	100 (31)	60 (30)	0.754		
Solid organ transplant	24 (7)	10 (5)	0.312		
Other	56 (17)	28 (14)	0.288		
Allogenic HCT	54 (17)	41 (25)	0.303		
AHRF episode					
Duration of symptoms prior to admission, *days*	2 (1–6)	3 (1–10)	0.027		
SOFA	5 (3–8)	6 (5–9)	<0.001	1.13 (1.07–1.20)	<0.0001
*Cause of AHRF, n (%)*			0.138		
Bacterial pneumonia	67 (21)	45 (22)			
Viral pneumonia	49 (15)	26 (13)			
Invasive fungal disease	36 (11)	28 (14)			
Disease related infiltrates	43 (13)	32 (16)			
Acute cardiogenic pulmonary edema	31 (10)	10 (5)			
Other	62 (19)	29 (14)			
Undetermined	33 (10)	31 (15)			
At ICU admission					
Respiratory rate, *min^−1^*	31 (26–36)	32 (28–38)	0.064		
Dyspnea-NRS	5 (3–7)	7 (5–8)	<0.001	1.19 (1.10–1.29)	<0.0001
Dyspnea-NRS ≥5*, n (%)*	191 (59)	159 (79)	<0.001		
Bilateral alveolar infiltrate on chest x-ray*, n (%)*	112 (35)	85 (42)	0.089		
PaO_2_/FiO_2_, *mmHg*	170 (118–264)	163 (112–270)	0.400		
PaCO_2_, *mmHg*	36 (30–43)	35 (29–43)	0.972		
Neutropenia*, n (%)*	58 (18)	50 (25)	0.062		
Non-invasive respiratory support, n (%)			0.485		
Conventional oxygen therapy	177 (55)	99 (49)			
Non-invasive ventilation (NIV)	45 (14)	36 (18)			
High flow oxygen therapy (HFO)	59 (18)	42 (21)			
NIV and HFO	40 (12)	24 (12)			
Organ support, n (%)					
Vasopressors	111 (35)	118 (59)	<0.001		
Renal replacement therapy	35 (11)	31 (15)	0.131		
Treatment limitation decision, n (%)	15 (5)	22 (11)	0.007		

Quantitative variables are reported as median (interquartile range [IQR]) qualitative variables are reported as frequency (percentages).

BMI, body mass index; COPD, chronic obstructive pulmonary disease; ECOG, eastern oncology study group; HCT, hematopoietic cells transplantation; AHRF, acute hypoxemic respiratory failure; SOFA, Sequential Organ Failure Assessment score; ICU, intensive care unit; dyspnea-NRS, numeric rating scale for dyspnea.

a ECOG was available in 466 patients.

### Factors associated with dyspnea severity on ICU admission

After adjustment for confounders and clustering effect, variables independently associated with dyspnea-NRS were underlying immune defect unrelated to hematological malignancy (estimate 0.59 ± standard deviation 0.30; P = 0.045), chronic heart failure (0.61 ± 0.024, P < 0.001), SOFA score (per point, 0.09 ± 0.03, P < 0.001) and respiratory rate (per breath per minute, 0.06 ± 0.01; p < 0.001). Of notice, the correlation between dyspnea and respiratory rate was weak (r = 0.25, p < 0.001).

## Discussion

The results of this large prospective international cohort study summarize as follows. In non-intubated immunocompromised patients admitted to the ICU for AHRF: (1) dyspnea is frequent and severe and is linked to the nature of the underlying cause of immunosuppression and to the severity of the current AHRF episode, (2) dyspnea-NRS ≥5 is associated with a higher risk of intubation, (3) dyspnea is associated with increased mortality. To the best of our knowledge, this is the largest study to investigate dyspnea in a population of non-intubated immunocompromised patients managed for AHRF.

With median value as high as 5, 84% of patients reporting a score of 3 and more and 76 % reporting a score of 4 or more, dyspnea intensity on ICU admission was high. As comparison, only 21% of patients admitted to the emergency room with acute or decompensated respiratory disease had dyspnea-NRS ≥4 [13]. Of note, because the present study is among the few that describe dyspnea in non-intubated patients with AHRF, we did not use the dyspnea-NRS cut-off of 3 or 4 that define significant dyspnea in intubated acutely il patients or in the palliative care setting. We rather describe dyspnea by using the quartiles value of dyspnea-NRS in our study population. Because dyspnea results from load capacity imbalance, it is a key symptom of AHRF. It is therefore not unexpected to observe frequent and severe dyspnea in our population. However, dyspnea was worse than what has been reported in unselected populations of non-intubated patients with AHRF [5,6]. The reason why dyspnea is worse in immunocompromised patients in unclear. This may involve the underlying cause of AHRF, disease related infiltration, invasive fungal infection, and other but still unknown diagnosis being much more frequent in immunocompromised hosts. Not only the high degree of dyspnea causes immediate suffering, it also contributes to the distressing recollections of patients following their ICU stay and to the pathogenesis of post-traumatic stress disorders [14,15]. Similar pain scores would constitute a clear indication for analgesia [16]. Dyspnea should be actively sought for in patients with AHRF, with routine use of a validated dyspnea scale, and treated specifically, in the same way as one would treat pain. It must be stressed that relief of dyspnea is an essential clinical mission that, like pain, is currently considered by some authors as a basic human right [17,18].

A major finding of the present study is that dyspnea-NRS ≥5 was independently associated with intubation and subsequent mechanical ventilation, along with severity score. This association between dyspnea and intubation has been previously observed in unselected populations of patients receiving either standard oxygen, high flow nasal cannula or non-invasive ventilation support for AHRF and in COVID-19 patients [[5], [6], [7]]. This association is not unexpected since dyspnea proceeds from respiratory system load capacity imbalance, which is also the main reasons for intubation. Because delayed intubation is associated with increased mortality in immunocompromised patients [19], physicians are looking for accurate predictors of future intubation that are easy to monitor [[20], [21], [22]]. Our results suggest that routinely monitoring dyspnea is a factor that should assist physicians in the intubation decision-making process and hence improve the management of immunocompromised patients admitted for AHRF. Of note, this is all the more important that usual markers of AHRF severity such as tachypnea and hypoxemia were not independently associated with intubation.

Dyspnea was associated with higher short-term and long-term mortality, independently of SOFA, a robust predictor of mortality. It suggests that dyspnea is a marker of severity that results from mechanisms that usual severity scores do not integrate, like respiratory system load capacity balance. It makes dyspnea a proxy for the severity of the underlying respiratory disease [23]. Previous studies have reported an association between dyspnea and hospital mortality in patients with suspected acute myocardial infarction [24,25], in those admitted for acute COPD exacerbation [26] and even in patients without previously diagnosed cardiopulmonary diseases [13,27,28]. Here, the relationship between dyspnea and mortality was almost linear, as previously reported in a cohort of 67362 consecutive hospital admissions [13]. To the best of our knowledge, this is the first study to report an association between dyspnea on ICU admission and a higher mortality rate in patients with AHRF and the first to report this association exclusively in immunocompromised patients [6].

The major strength of our study is the prospective multicenter and multinational design, which gives robust external validity to the results. This study has limitations. First, we chose to quantify dyspnea by means of a numeric rating scale rather than a visual analogue scale or a Borg scale. These are the three instruments most commonly used to measure dyspnea in the ICU [29]. There are strong correlations between these scales, and they have all demonstrated validity and reliability in critically ill patients [[30], [31], [32], [33]]. Second, we quantified dyspnea only on admission to the ICU. A longitudinal analysis based on multiple repeated measurements would provide additional insight [34]. Third, patients were not systematically assessed for delirium, which may impact self-reporting of respiratory comfort. However, respiratory comfort was not collected in patients who were unable to provide clear and coherent answers and these patients were not included in the study (see Fig. 1). Fourth, dyspnea was not measured in all patients, which introduces a selection bias. Fifth, intubation criteria were not predefined. Sixth, the fact that patients were included only in centres with extensive experience in the management of critically ill immunocompromised patients lowers the external validity of this study. Seventh, data were collected 10 years ago, when high flow oxygen was not used as frequently as today. Therefore, our results cannot fully translate to current practice.

*In conclusion,* in this multicenter cohort, dyspnea was associated with a worse outcome in immunocompromised patients admitted to the ICU for AHRF. Dyspnea seemed to be a warning signs in these patients. This symptom being easy to detect and quantify at bedside, dyspnea should be among the variables that are collected in these patients. In addition, because dyspnea generates immediate suffering and delayed distressing recollections and post-traumatic stress disorders, a systematic measurement and recording of dyspnea would be fully justified. The present study paves the way for future studies aimed at monitoring dyspnea in immunocompromised patients with AHRF. It also paves the way for interventional studies that would evaluate the impact on patient outcome of pharmacological and non-pharmacological therapies to relieve dyspnea.

## Author contributions

Alexandre Demoule, Elie Azoulay and Michael Darmon are responsible for study design, data acquisition, data analysis, data interpretation, manuscript writing, manuscript review, manuscript approval for publishing, and was accountable for all aspects of the work. Maxens Decavèle, Sangeeta Mehta, Philippe R. Bauer, Victoria Metaxa, Frédéric Pène, Christophe Girault, Laveena Munshi, Fabio Silvio Taccone, Massimo Antonelli, Francois Barbier, Andreas Barratt-Due, Gaston Burghi, Emmanuel Canet, Achille Kouatchet, Virginie Lemiale, Ignacio Martin-Loeches, Djamel Mokart, Anne-Sophie Moreau, Luca Montini, Peter Pickkers, Jordi Rello, Peter Schellongowski, Nicolas Terzi, Miia Valkonen and Andry van de Louw were responsible for patients enrollment, interpretation of data, and drafting the manuscript. All authors approved the final version of the manuscript.

## Consent for publication

Not applicable.

## Ethics approval and consent to participate

The study was approved by the institutional review board of each institution in accordance with local ethics regulation (Table E1 in the Online Supplement).

## Disclosure of funding received for the work from any of the following organizations

Fondation du Souffle, France.

## Availability of data and materials

The datasets used and/or analysed during the current study are available from the corresponding author on reasonable request.

## Declaration of competing interest

Alexandre Demoule reports grants from the French Ministry of Health, Respinor, Liberate Medical, Bio Aegis/Ergomed, consulting fees from Liberate Medical, Respinor, SAT Lutech, Black Fur Medical, WK Health Medical Researc, Philips, payment of honoraria for lecture from Fisher&Paykel, support for attending meeting, outside the submitted work. Maxens Decavèle reports payment of honoraria for lecture Fisher&Paykel, travel fees from Fisher&Paykel, outside the submitted work. Sangeeta Mehta reports no conflict of interest. Philippe R. Bauer reports no conflict of interest. Victoria Metaxa reports payment of honoraria for lecture from Gilead, outside the submitted work. Frédéric Pène reports consulting fees from Gilead, Miunnipharma, payment of honoraria for lecture from Gilead, travel fees from Gilead, outside the submitted work. Christophe Girault reports payment of honoraria for lecture Fisher&Paykel, travel fees from Fisher&Paykel, Asten Santé, teaching equipment from Resmed, Fisher&Paykel, outside the submitted work. Laveena Munshi reports no conflict of interest. Fabio Silvio Taccone reports no conflict of interest. Massimo Antonelli reports grants from GE and Fisher & Paykel, consulting fees from Menarini, Grifols, outside the submitted work. Francois Barbier reports no conflict of interest. Andreas Barratt-Due reports no conflict of interest. Gaston Burghi reports no conflict of interest. Emmanuel Canet reports payment of honoraria for lectures from Gilead, Shionogi, Sanofi-Genzyme, support for attending meeting from Gilead, Shionogi, Sanofi-Genzyme, outside the submitted work. Achille Kouatchet reports no conflict of interest. Virginie Lemiale reports no conflict of interest. Ignacio Martin-Loeches reports no conflict of interest. Djamel Mokart reports no conflict of interest. Anne-Sophie Moreau, reports no conflict of interest. Luca Montini reports no conflict of interest. Peter Pickkers reports no conflict of interest. Jordi Rello reports no conflict of interest. Peter Schellongowski reports payment of honoraria for lecture from Getinge, Fresenius Medical, outside the submitted work. Nicolas Terzi reports payment of honoraria for lecture from Fisher&Paykel, support for attending meeting from Gilead, outside the submitted work. Miia Valkonen reports no conflict of interest. Andry van de Louw reports no conflict of interest. E Azoulay reports grants from Fisher&Paykel, MSD, payment of honoraria for lecture from Gilead, Alexion, Fisher&Paykel, Baxter, outside the submitted work. M Darmon reports grants and personal fees from MSD, personal fees and non-financial support from Gilead-Kite, personal fees from Astellas, outside the submitted work.
