# Supplementary material for: Dyspnea as a marker of prognosis in immunocompromised patients with acute respiratory failure
Source: Ann Intensive Care. 2026 May 19;16:100091. doi: 10.1016/j.aicoj.2026.100091 (PMC13241896; doi:10.1016/j.aicoj.2026.100091)
Supplement: Supplementary file 1 [file mmc1.docx]

**Dyspnea on admission as a marker of prognosis**

**in immunocompromised patients with acute respiratory failure: A secondary analysis of the Efraim prospective observational study**

**Online Supplement**

**Table E1. List of the Institutional Review Boards (IRB) that reviewed the study and IRB approval number**

**Table E2. STROBE Statement—Checklist of items that should be included in reports of cohort studies**

**Table E1. List of the Institutional Review Boards (IRB) that reviewed the study and IRB approval number**

| **Country** | **City** | **Institution** | **Name of IRB** | **IRB number** |
| --- | --- | --- | --- | --- |
| Austria | Gratz | Medical University of Gratz | Medical University of Gratz | 28-125 ex 15/16 |
|  | Vienna | Department of Medicine I | Medical University of Vienna | 1823/2915 |
|  | Vienna | Department of Medicine II | Medical University of Vienna | 1823/2915 |
|  | Vienna | Department of Medicine III | Medical University of Vienna | 1823/2915 |
|  | Vienna | Department of Thoracic Surgery | Medical University of Vienna | 1823/2915 |
| Belgium | Bruxelles | Hôpital Erasme, ULB | Comité d’Ethique Erasme-ULB | P2018/128 |
|  | Bruxelles | Institut Jules Bordet | Comité Ethique de l'Insitut Jules Bordet | CE-2470 |
|  | Ghent | Ghent University Hospital | Universitair Ziekenhuis Gent | 2015/1352 |
| Brazil | Barretos | Hospital de Câncer de Barretos | Instituto D'Or De Pesquisa | 1.461.224 |
|  | Maranhao | Hospital de Câncer do Maranhao | Instituto D'Or De Pesquisa | 1.461.224 |
|  | Porte Allegre | Hospital Santa Rita, Santa Casa de Misericordia | Instituto D'Or De Pesquisa | 1.461.224 |
|  | Rio De Janeiro | D’Or Institute for Research and Education | Instituto D'Or De Pesquisa | 1.461.224 |
|  | Rio De Janeiro | Hospital Copa d’Or | Instituto D'Or De Pesquisa | 1.461.224 |
|  | Rio De Janeiro | Hospital GetulioVargas | Instituto D'Or De Pesquisa | 1.461.224 |
|  | São Paulo | Hospital Israelita Albert Einstein | Instituto D'Or De Pesquisa | 1.461.224 |
| Canada | Toronto | Universy of Toronto | Research Ethics Board, Mount Sinai Hospital | 15-0264-C |
| Czech republic | Pilsen | University Hospital in Pilsen | Ethics Committee of the University Hospital in Pilsen | NA (2015-12-3) |
|  | Pragues | 1st Faculty of Medicine, Charles University in Prague and General University Hospital | General University Hospital, Prague | 1859/15 S-IV |
| Denmark | Copenhagen | Rigshospitalet, University of Copenhagen | Danish Health Authority | 3-3013-1268 |
|  | Herlev | Herlev University Hospital | Danish Health Authority | 3-3013-1268 |
|  | Odense | Odense UniversityHospital, University of South Denmark | Danish Health Authority | 3-3013-1268 |
|  | Sønderborg | Intensive Care Department, University of Southern Denmark | Danish Health Authority | 3-3013-1268 |
| France | Angers | Centre hopsitalier Régional | French Intensive Care Society | CE SRLF 15-34 |
|  | Caen | CHU de Caen | French Intensive Care Society | CE SRLF 15-34 |
|  | Étampes | Centre Hospitalier Sud Essonne | French Intensive Care Society | CE SRLF 15-34 |
|  | Grenoble | CHU Grenoble Alpes | French Intensive Care Society | CE SRLF 15-34 |
|  | La Roche sur Yon | Centre Hospitalier de Vendée | French Intensive Care Society | CE SRLF 15-34 |
|  | Lille | Centre hospitalier universitaire |  |  |
|  | Lyon | Centre Hospitalier Lyon-Sud | French Intensive Care Society | CE SRLF 15-34 |
|  | Marseille | Institut Paoli Calmette | French Intensive Care Society | CE SRLF 15-34 |
|  | Montpellier | CHU de Montpellier | French Intensive Care Society | CE SRLF 15-34 |
|  | Nantes | Hôtel Dieu | French Intensive Care Society | CE SRLF 15-34 |
|  | Orléans | La Source Hospital | French Intensive Care Society | CE SRLF 15-34 |
|  | Paris | Hôpital Cochin | French Intensive Care Society | CE SRLF 15-34 |
|  | Paris | Hôpital Cochin | French Intensive Care Society | CE SRLF 15-34 |
|  | Paris | Hôpital Pitié-Salpétrière | French Intensive Care Society | CE SRLF 15-34 |
|  | Paris | Hôpital Saint-Louis | French Intensive Care Society | CE SRLF 15-34 |
|  | Roubaix | Centre Hospitalier Victor Provo | French Intensive Care Society | CE SRLF 15-34 |
|  | Rouen | Rouen University Hospital | French Intensive Care Society | CE SRLF 15-34 |
|  | Saint Etienne | CHU de Saint-Etienne | French Intensive Care Society | CE SRLF 15-34 |
|  | Versailles | Hôpital André Mignot | French Intensive Care Society | CE SRLF 15-34 |
|  | Villejuif | Institut Gustave Roussy | French Intensive Care Society | CE SRLF 15-34 |
| Finland | Helsinki | Helsinki University Hospital | Helsingi Ja Uudenmaan Sairaanhoitopiiri, Helsinski | 180 |
|  | Tampere | Tampere University Hospital | Helsingi Ja Uudenmaan Sairaanhoitopiiri, Helsinski | 180 |
| Irland | Dublin | Mater Misericordia | Ethic committee St James's/Tallaght IRB | Waived consent - No number provided |
|  | Dublin | Saint-James Hopsital | Ethic committee St James's/Tallaght IRB | Waived consent - No number provided |
| Italy | Foggia | Ospedali Riuniti | Comitato Etico - Ospedali Riuniti | 53/CE/2016 |
|  | Roma | Policlinica Gemelli | Comitato Etico della Fondazione Policlinico Gemelli | 00139947/16 |
| Netherlands | Amsterdam | University Medical Center | VU Universirt Medical Center | FWA00017598 |
|  | Groningen | University Medical Center | VU Universirt Medical Center | FWA00017598 |
|  | Maastricht | University Medical Center | VU Universirt Medical Center | FWA00017598 |
|  | Nijmegen | Radboud University Medical Center | VU Universirt Medical Center, | FWA00017598 |
| Norway | Oslo | Oslo University Hospital | REK Sør-Øst (Regional Ethical commitee south-east) | 2015/1872A |
|  | Trondheim | St. Olavs Hospital | REK Sør-Øst (Regional Ethical commitee south-east) | 2015/1872A |
| Spain | Barcelona | Universitat Autonòma de Barcelona | CEIC Vall d'Hebron | PR(AG)260/2015 |
|  | Barcelona | Bellvitge | CEIC Bellvitge | PR312/15 |
|  | Palma | Hospital Son Llatzer | CEIC Vall d'Hebron | PR(AG)260/2015 |
|  | Santiago de compostella | Santiago de compostella Hospital | Comite de Etica de la Investigacion de Santiago-Lugo | 2015/611 |
| United Kingdom | London | King's college Hospital | Kings College Hospital | KCC06082015ACU |
| Uruguay | Montevideo | Hospital Maciel | Comité de Ética de la Investigación del Hospital Maciel | 2015-10-21 |
| United States of America | Hershey | Pennsylvania State University | Pennsylvania State University | 3315 |
|  | Rochester | Mayo clinic | Mayo Clinic Institutional Review Board | 15-006975 |

**Table E2. STROBE Statement—checklist of items that should be included in reports of observational studies**

|  | Item No. | Recommendation | Page  No. | Relevant text from manuscript |
| --- | --- | --- | --- | --- |
| **Title and abstract** | 1 | (*a*) Indicate the study’s design with a commonly used term in the title or the abstract | 6 | In the abstract: “Secondary analysis of the Efraim study, a prospective multinational cohort study” |
|  |  | (*b*) Provide in the abstract an informative and balanced summary of what was done and what was found | 6 | Dyspnea was quantified by a numeric rating scale (dyspnea-NRS) from zero to 10. Factors associated with dyspnea-NRS were assessed with linear regression. Hierarchical model was used to assess factors independently associated with invasive mechanical ventilation (intubation) and hospital mortality.  547 patients were included. On ICU admission, median dyspnea-NRS was 5 (interquartile range 4‒7). Variables independently associated with dyspnea-NRS were underlying immune defect unrelated to hematological malignancy, chronic heart failure, high SOFA score and respiratory rate. Intubation rate was 41 %. Variables independently associated with intubation were dyspnea-NRS ≥5 (odds ratio [OR] 2.61, p<0.001), high SOFA (OR per point 1.10, p=0.006) and fungal infection (OR 2.02, p=0.020)., while respiratory rate and PaO_2_/FiO_2_ were not. Hospital mortality was 37 %. Variables independently associated with hospital mortality were age (OR per year 1.02, P=0.009), SOFA score (OR per point, 1.13, P<0.001) and dyspnea-NRS (OR per point 1.19, P<0.001). |
| Introduction | | | |  |
| Background/rationale | 2 | Explain the scientific background and rationale for the investigation being reported | 7 | See introduction |
| Objectives | 3 | State specific objectives, including any prespecified hypotheses | 7 | Here, we hypothesized that, in non-intubated immunocompromised patients managed for AhRF, dyspnea severity on ICU admission is associated with higher risk of intubation and mortality. The primary aim was to assess whether dyspnea severity on admission predicts the need for intubation. Secondary aims were to identify factors associated with higher dyspnea severity and to evaluate the association between dyspnea severity and hospital mortality. |
| Methods | | | |  |
| Study design | 4 | Present key elements of study design early in the paper | 8 | First sentence of methods: “This is a preplanned secondary analysis of the Efraim multinational, observational prospective cohort study on immunocompromised patients admitted in the ICU for AhRF. This initiative from the Nine-I (Caring for critically ill immunocompromised patients) study group has included patients from 68 ICUs in 16 countries.” |
| Setting | 5 | Describe the setting, locations, and relevant dates, including periods of recruitment, exposure, follow-up, and data collection | 8 and 9 | each participating ICUs prospectively included patients between November 2015 and July 2016. each participating ICUs prospectively included patients between November 2015 and July 2016.  Demographic data and medical history collected consisted of: age, gender, body mass index, performance status, respiratory or cardiac comorbidity, cause of immunosuppression and neutropenia. The precipitating factor of AhRF was recorded. Data on the current AhRF episode included Sequential Organ Failure Assessment score (SOFA) [11], respiratory rate, respiratory comfort, chest radiography, arterial blood gas and initial oxygenation strategy. Dyspnea, termed “comfort with breathing” on the case report form, was assessed with a patient self-reported numerical rating scale (dyspnea-NRS) from zero (no respiratory discomfort) to 10 (worst possible respiratory comfort). Of note, this question did not target discomfort associated with the respiratory interface (mask, nasal canulae, etc.) or the discomfort associated with any non-invasive respiratory support delivered to the patient (non-invasive ventilation, high flow nasal canulae, etc.). No trigger question regarding dyspnea absence or presence (yes-no question) was asked to the patients. Need for intubation, catecholamine and renal replacement therapy was recorded, as well as occurrence of Acute Respiratory Distress Syndrome according the Berlin definition [12]. ICU, in-hospital and 90-day mortality were recorded, as well as ICU and hospital length of stay. Patient code status on ICU admission (full code or treatment limitation decision) was recorded. |
| Participants | 6 | (*a*) *Cohort study*—Give the eligibility criteria, and the sources and methods of selection of participants. Describe methods of follow-up  *Case-control study*—Give the eligibility criteria, and the sources and methods of case ascertainment and control selection. Give the rationale for the choice of cases and controls  *Cross-sectional study*—Give the eligibility criteria, and the sources and methods of selection of participants | 8, 9 | Inclusion criteria were age ≥ 18 years, acute hypoxemic respiratory failure (PaO_2_ < 60 mmHg or SpO_2_ < 90% on room air, or tachypnea > 30/min, or labored breathing or respiratory distress or dyspnea at rest or cyanosis), need for more than 6 L/min oxygen, respiratory symptom duration less than 72 h and non-AIDS-related immune deficiency defined as hematologic malignancy or solid tumor (active or in remission for less than 5 years, including recipients of hematopoietic cell transplantation), solid organ transplant, long-term (> 30 days) or high-dose (> 1 mg/kg/day) steroids, or any immunosuppressive drug for more than 30 days. Patients with postoperative acute respiratory failure (within 6 days of surgery), those admitted after a cardiac arrest, patients admitted only to secure bronchoscopy, and patients/surrogates who declined study participation were not included.  Study investigators completed a standardized paper case report form that was eventually sent to the coordinating center in Paris. |
|  |  | (*b*) *Cohort study*—For matched studies, give matching criteria and number of exposed and unexposed  *Case-control study*—For matched studies, give matching criteria and the number of controls per case |  | Not applicable |
| Variables | 7 | Clearly define all outcomes, exposures, predictors, potential confounders, and effect modifiers. Give diagnostic criteria, if applicable | 9 | The primary outcome was intubation during ICU stay. Secondary outcomes were ICU, hospital and 90-day mortality, as well as ICU and hospital length of stay. |
| Data sources/ measurement | 8* | For each variable of interest, give sources of data and details of methods of assessment (measurement). Describe comparability of assessment methods if there is more than one group | *9* | Study investigators completed a standardized paper case report form that was eventually sent to the coordinating center in Paris.  Demographic data and medical history collected consisted of: age, gender, body mass index, performance status, respiratory or cardiac comorbidity, cause of immunosuppression and neutropenia. The precipitating factor of AhRF was recorded. Data on the current AhRF episode included Sequential Organ Failure Assessment score (SOFA) [11], respiratory rate, respiratory comfort, chest radiography, arterial blood gas and initial oxygenation strategy. Dyspnea, termed “comfort with breathing” on the case report form, was assessed with a patient self-reported numerical rating scale (dyspnea-NRS) from zero (no respiratory discomfort) to 10 (worst possible respiratory comfort). Of note, this question did not target discomfort associated with the respiratory interface (mask, nasal canulae, etc.) or the discomfort associated with any non-invasive respiratory support delivered to the patient (non-invasive ventilation, high flow nasal canulae, etc.). No trigger question regarding dyspnea absence or presence (yes-no question) was asked to the patients. Need for intubation, catecholamine and renal replacement therapy was recorded, as well as occurrence of Acute Respiratory Distress Syndrome according the Berlin definition [12]. ICU, in-hospital and 90-day mortality were recorded, as well as ICU and hospital length of stay. Patient code status on ICU admission (full code or treatment limitation decision) was recorded. |
| Bias | 9 | Describe any efforts to address potential sources of bias | 10, 11 | To assess linear relationship of dyspnea with invasive ventilation and mortality, a gam model was performed and resulting spline are reported. Based on the spline, we decided to analyze association between dyspnea and mechanical ventilation as a binary variable (dyspnea-NRS<5 vs. ≥5) and association of dyspnea with mortality as a continuous variable.  To assess linear relationship of dyspnea with invasive ventilation and mortality, a gam model was performed and resulting spline are reported. Based on the spline, we decided to analyze association between dyspnea and mechanical ventilation as a binary variable (dyspnea-NRS<5 vs. ≥5) and association of dyspnea with mortality as a continuous variable.  We used conditional stepwise regression with 0.2 as the critical P-value for entry into the model, and 0.1 as the P-value for removal. It was planned a priori to force dyspnea in the model should this variable not be selected. Interactions and correlations between the explanatory variables were carefully checked. Continuous variables for which log-linearity was not confirmed were transformed into categorical variables according to median or IQR. The final models were assessed by calibration, discrimination and relevancy. Residuals were plotted, and the distributions inspected. A hierarchical model was then performed using variables previously selected along with center as random effect on the intercept. This model adjusting for clustering effect was planned a priori to be main result of the analysis. Same validation methods were used as previously. Adjusted odds ratios (OR) of variables present in the final model are presented with their 95% confidence intervals (CI). |
| Study size | 10 | Explain how the study size was arrived at | 10 | Convenient sample size |

Continued on next page

| Quantitative variables | 11 | Explain how quantitative variables were handled in the analyses. If applicable, describe which groupings were chosen and why | 10 | Dyspnea-NRS on admission was used to identify four groups of patients according to quartile: zero to 3, 4 to 5, 6 to 7 and 8 to 10. Hierarchical models were used to assess factors independently associated with dyspnea, intubation and mortality. To assess variables associated with dyspnea severity on ICU admission, a correlation plot was performed between dyspnea and continuous variables. Then a linear regression was performed, dyspnea being the variable of interest. Conditional stepwise regression with 0.2 as the critical P-value for entry into the model, and 0.1 as the P-value for removal. Dyspnea reporting being liable to vary from center to center, the final model was a mixed model with center as a random effect on the intercept. |
| --- | --- | --- | --- | --- |
| Statistical methods | 12 | (*a*) Describe all statistical methods, including those used to control for confounding | 10 and 11 | Logistic regression was performed to assess variables associated with need for intubation over ICU stay and with hospital mortality. To assess linear relationship of dyspnea with invasive ventilation and mortality, a gam model was performed and resulting spline are reported. Based on the spline, we decided to analyze association between dyspnea and mechanical ventilation as a binary variable (dyspnea-NRS<5 vs. ≥5) and association of dyspnea with mortality as a continuous variable.  We used conditional stepwise regression with 0.2 as the critical P-value for entry into the model, and 0.1 as the P-value for removal. It was planned a priori to force dyspnea in the model should this variable not be selected. Interactions and correlations between the explanatory variables were carefully checked. Continuous variables for which log-linearity was not confirmed were transformed into categorical variables according to median or IQR. The final models were assessed by calibration, discrimination and relevancy. Residuals were plotted, and the distributions inspected. A hierarchical model was then performed using variables previously selected along with center as random effect on the intercept. This model adjusting for clustering effect was planned a priori to be main result of the analysis. Same validation methods were used as previously. Adjusted odds ratios (OR) of variables present in the final model are presented with their 95% confidence intervals (CI).  Kaplan-Meier graphs were used to express the probability of death from inclusion to hospital discharge, censored at day 90 and were compared across groups by the log rank test.  A p value <0.05 was considered significant. Statistical analyses were performed with IBM SPSS Statistics, version 20.0 (IBM SPSS Inc., Chicago, IL, USA) and with R statistical software, version 3.4.4 (available online at http://www.r-project.org/) and packages ‘mgcv’, ‘survival’, ‘lme4’,and ’lmerTest’. |
|  |  | (*b*) Describe any methods used to examine subgroups and interactions |  | NA |
|  |  | (*c*) Explain how missing data were addressed |  |  |
|  |  | (*d*) *Cohort study*—If applicable, explain how loss to follow-up was addressed  *Case-control study*—If applicable, explain how matching of cases and controls was addressed  *Cross-sectional study*—If applicable, describe analytical methods taking account of sampling strategy |  | Not included in the final analysis |
|  |  | (*e*) Describe any sensitivity analyses |  | NA |
| Results | | | | |
| Participants | 13* | (a) Report numbers of individuals at each stage of study—eg numbers potentially eligible, examined for eligibility, confirmed eligible, included in the study, completing follow-up, and analysed | 11 | During the study period, 1611 immunocompromised patients were admitted AhRF, among whom 596 were intubated on ICU admission. Data on initial respiratory support were missing in 100. Among the 915 patients who were not intubated on ICU admission, dyspnea was not recorded in 368 patients. A total of 547 patients were included in this analysis (Figure 1). Table 1 displays the main characteristics of these patients. |
|  |  | (b) Give reasons for non-participation at each stage | 11 | During the study period, 1611 immunocompromised patients were admitted AhRF, among whom 596 were intubated on ICU admission. Data on initial respiratory support were missing in 100. Among the 915 patients who were not intubated on ICU admission, dyspnea was not recorded in 368 patients. A total of 547 patients were included in this analysis (Figure 1). Table 1 displays the main characteristics of these patients. |
|  |  | (c) Consider use of a flow diagram | Figure 1 | See Figure 1 for flow diagram |
| Descriptive data | 14* | (a) Give characteristics of study participants (eg demographic, clinical, social) and information on exposures and potential confounders | 12 | During the study period, 1611 immunocompromised patients were admitted AhRF, among whom 596 were intubated on ICU admission. Data on initial respiratory support were missing in 100. Among the 915 patients who were not intubated on ICU admission, dyspnea was not recorded in 368 patients. A total of 547 patients were included in this analysis (Figure 1). Table 1 displays the main characteristics of these patients. |
|  |  | (b) Indicate number of participants with missing data for each variable of interest | 22, 23, 24, 25 | See Tables 1, 2, 3, 4. |
|  |  | (c) *Cohort study*—Summarise follow-up time (eg, average and total amount) |  | Not applicable |
| Outcome data | 15* | *Cohort study*—Report numbers of outcome events or summary measures over time | *12, 13* | A hierarchical model was then performed using variables previously selected along with center as random effect on the intercept. This model adjusting for clustering effect was planned a priori to be main result of the analysis.  ICU mortality was available in all patients. Hospital mortality and 90-day mortality were available in 522 and 452 patients, respectively. ICU, hospital and 90-day mortality were 24 %, 37 % and 42 %, respectively. On univariate analysis, higher dyspnea-NRS was associated with higher ICU, hospital and 90-day mortality (Table 2). Dyspnea was not associated with ICU or hospital length of stay and was not associated with the use of vasopressors or renal replacement therapy after intubation (Table 2). |
|  |  | *Case-control study—*Report numbers in each exposure category, or summary measures of exposure |  | *NA* |
|  |  | *Cross-sectional study—*Report numbers of outcome events or summary measures |  | *NA* |
| Main results | 16 | (*a*) Give unadjusted estimates and, if applicable, confounder-adjusted estimates and their precision (eg, 95% confidence interval). Make clear which confounders were adjusted for and why they were included | 12 and Table 2  13 and Table 4 | After adjustment for confounders and clustering effect, three variables were independently associated with intubation: dyspnea-NRS ≥5 (OR 2.61, 95% CI 1.74‒3.91, P<0.001), SOFA (OR per point 1.10, 95% CI 1.04‒1.17, P=0.006), and AhRF etiology, with fungal infection being associated with a higher risk of intubation (OR 2.02, 95% CI 1.03‒3.96, P=0.02, bacterial infection as reference etiology). When forced into the final model, respiratory rate, PaO_2_/FiO_2_ and the type of respiratory support were neither selected nor changed the final model.  The association between dyspnea and hospital mortality was linear (Figure 2, B). The comparison between survivors and non survivors at hospital discharge is shown in Table 4. After adjustment for confounders and clustering effect, three variables were independently associated with higher hospital mortality: age (OR per year 1.02, 95%CI 1.00‒1.03; P=0.009), severity as assessed by the SOFA score (OR per point, 1.13, 95%CI 1.07‒1.20; P<0.0001) and dyspnea-NRS (OR per point 1.19, 95%CI 1.10-1‒29; P<0.0001). When forced in the final model, performance status, the cause of AhRF and the type of respiratory support were neither selected nor changed the final model. |
|  |  | (*b*) Report category boundaries when continuous variables were categorized |  |  |
|  |  | (*c*) If relevant, consider translating estimates of relative risk into absolute risk for a meaningful time period |  | NA |

Continued on next page

| Other analyses | 17 | Report other analyses done—eg analyses of subgroups and interactions, and sensitivity analyses |  | NA |
| --- | --- | --- | --- | --- |
| Discussion | | | | |
| Key results | 18 | Summarise key results with reference to study objectives | 13, 14 | The results of this large prospective international cohort study summarize as follows. In non-intubated immunocompromised patients admitted to the ICU for AhRF: 1) dyspnea is frequent and severe and is linked to the nature of the underlying cause of immunosuppression and to the severity of the current AhRF episode, 2) dyspnea-NRS ≥5 is associated with a higher risk of intubation, 3) dyspnea is associated with increased mortality. To the best of our knowledge, this is the largest study to investigate dyspnea in a population of non-intubated immunocompromised patients managed for AhRF. |
| Limitations | 19 | Discuss limitations of the study, taking into account sources of potential bias or imprecision. Discuss both direction and magnitude of any potential bias | 15, 16 | This study has limitations. First, we chose to quantify dyspnea by means of a numeric rating scale rather than a visual analogue scale or a Borg scale. These are the three instruments most commonly used to measure dyspnea in the ICU [29]. There are strong correlations between these scales, and they have all demonstrated validity and reliability in critically ill patients [30–33]. Second, we quantified dyspnea only on admission to the ICU. A longitudinal analysis based on multiple repeated measurements would provide additional insight [34]. Third, patients were not systematically assessed for delirium, which may impact self-reporting of respiratory comfort. However, respiratory comfort was not collected in patients who were unable to provide clear and coherent answers and these patients were not included in the study (see Figure 1). Fourth, dyspnea was not measured in all patients, which introduces a selection bias. |
| Interpretation | 20 | Give a cautious overall interpretation of results considering objectives, limitations, multiplicity of analyses, results from similar studies, and other relevant evidence | 16 | in this multicenter cohort, dyspnea was associated with a worse outcome in immunocompromised patients admitted to the ICU for AhRF. Dyspnea seemed to be a warning signs in these patients. |
| Generalisability | 21 | Discuss the generalisability (external validity) of the study results | 16 | This symptom being easy to detect and quantify at bedside, dyspnea should be among the variables that are collected in these patients. In addition, because dyspnea generates immediate suffering and delayed distressing recollections and post-traumatic stress disorders, a systematic measurement and recording of dyspnea would be fully justified. |
| Other information | |  | | |
| Funding | 22 | Give the source of funding and the role of the funders for the present study and, if applicable, for the original study on which the present article is based | 5 | Fondation du Souffle  This is a preplanned secondary analysis of the Efraim multinational, observational prospective cohort study on immunocompromised patients admitted in the ICU for AhRF. This initiative from the Nine-I (Caring for critically ill immunocompromised patients) study group has included patients from 68 ICUs in 16 countries. Participating physicians and teams have extensive experience in the management of various groups of critically ill immunocompromised patients. The full Efraim protocol and results have been published elsewhere [2]. |

*Give information separately for cases and controls in case-control studies and, if applicable, for exposed and unexposed groups in cohort and cross-sectional studies.

**Note:** An Explanation and Elaboration article discusses each checklist item and gives methodological background and published examples of transparent reporting. The STROBE checklist is best used in conjunction with this article (freely available on the Web sites of PLoS Medicine at http://www.plosmedicine.org/, Annals of Internal Medicine at http://www.annals.org/, and Epidemiology at http://www.epidem.com/). Information on the STROBE Initiative is available at www.strobe-statement.org.
